# In Silico Study of the Resistance to Organophosphorus Pesticides Associated with Point Mutations in Acetylcholinesterase of Lepidoptera: *B*. *mandarina*, *B*. *mori*, *C*. *auricilius*, *C*. *suppressalis*, *C*. *pomonella*, *H*. *armígera*, *P*. *xylostella*, *S*. *frugiperda,* and *S*. *litura*

**DOI:** 10.3390/ijms20102404

**Published:** 2019-05-15

**Authors:** Francisco Reyes-Espinosa, Domingo Méndez-Álvarez, Miguel A. Pérez-Rodríguez, Verónica Herrera-Mayorga, Alfredo Juárez-Saldivar, María A. Cruz-Hernández, Gildardo Rivera

**Affiliations:** 1 Laboratorio de Biotecnología Farmacéutica, Centro de Biotecnología Genómica, Instituto Politécnico Nacional, Reynosa 88710, Mexico; frelibi@hotmail.com (F.R.-E.); doomadv@hotmail.com (D.M.-Á.); veronica_qfb@hotmail.com (V.H.-M.); ajuarezs1500@gmail.com (A.J.-S.); tonitacruz@gmail.com (M.A.C.-H.); 2 Departamento de Botánica, Universidad Autónoma Agraria Antonio Narro, Saltillo 25315, Mexico; miguel_cbg@hotmail.com; 3 Departamento de Ingeniería Bioquímica, Unidad Académica Multidisciplinaria Mante, Universidad Autónoma de Tamaulipas, Mante 89840, Mexico

**Keywords:** acetylcholinesterase, resistance, organophosphorus, pesticides, molecular modeling, lepidopterous, insects

## Abstract

An in silico analysis of the interaction between the complex-ligands of nine acetylcholinesterase (AChE) structures of Lepidopteran organisms and 43 organophosphorus (OPs) pesticides with previous resistance reports was carried out. To predict the potential resistance by structural modifications in Lepidoptera insects, due to proposed point mutations in AChE, a broad analysis was performed using computational tools, such as homology modeling and molecular docking. Two relevant findings were revealed: (1) Docking results give a configuration of the most probable spatial orientation of two interacting molecules (AChE enzyme and OP pesticide) and (2) a predicted Δ*G*_b_. The mutations evaluated in the form 1 acetylcholinesterase (AChE-1) and form 2 acetylcholinesterase (AChE-2) structures of enzymes do not affect in any way (there is no regularity of change or significant deviations) the values of the binding energy (Δ*G*_b_) recorded in the AChE–OPs complexes. However, the mutations analyzed in AChE are associated with a structural modification that causes an inadequate interaction to complete the phosphorylation of the enzyme.

## 1. Introduction

A great diversity of organisms belonging to the order Lepidoptera is of great economic interest. As a study model, nine structures of Lepidoptera acetylcholinesterase (AChE) were treated: *Bombyx mandarina*, *Bombyx mori*, *Chilo auricilius*, *Chilo suppressalis*, *Cydia pomonella*, *Helicoverpa armígera*, *Plutella xylostella*, *Spodoptera frugiperda*, and *Spodoptera litura*. The mulberry silkworm, *B. mori*, is a Lepidopteran insect of great economic importance because of its use in natural silk fiber production and because it is a valuable insect model that has greatly enhanced our understanding of the biology of insects, including many agricultural pests. This insect is also often used for the production of recombinant eukaryotic proteins or as a model organism for pest control studies. The life cycle of the mulberry silkworm is well described; its genome was sequenced in 2004 [1]. *B. mandarina Moore* (Lepidoptera: Bombycidae) is an endangered wild Indian mulberry silkworm species [2]. The striped rice stem borer, *C. suppressalis*, is one of the most important rice pests in East Asia, India, and Indonesia. The main host plant of *C. suppressalis* is rice, maize, and many wild hosts [3]. The cotton bollworm, *H. armigera*, causes serious losses, in particular to cotton, tomatoes, and maize. The most important crop hosts, in which *H. armigera* is a major pest, are cotton, pigeonpea, chickpea, tomato, sorghum, and cowpea; other hosts include groundnut, okra, peas, field beans (*Lablab* spp.), soybeans, lucerne, *Phaseolus* spp., other Leguminosae, tobacco, potatoes, maize, flax, a number of fruits (Prunus, Citrus), forest trees, and a range of vegetable crops [4]. The resistance to pyrethroids in *H. armigera* can be conferred through three separate mechanisms: Detoxification by mixed function oxidases (metabolic resistance), nerve insensitivity, and delayed penetration [5]. The diamondback moth (DBM) in Mexico, *P. xylostella*, is one of the most studied insect pests in the world, yet it is among the ‘leaders’ of the most difficult pests to control. The DBM is a highly invasive species and it has shown resistance to almost every insecticide. The natural host plant range of the DBM is limited to Brassicaceae (also called Cruciferae), which is characterized by having glucosinolates, which are sulfur-containing secondary plant compounds. Cruciferous vegetables (such as cauliflower, cabbage, garden cress, bok choy, broccoli, and similar green leaf vegetables) are crop species that are cultivated for food production, and their weeds serve as alternate hosts. Some populations of DBM have also been found to infest non-cruciferous plants [6]. The tobacco caterpillar, *S. litura*, is one of the most important insect pests of agricultural crops in the Asian and African tropics. Among the main crop species attacked by *S. litura* in the tropics are *Colocasia esculenta*, cotton, flax, groundnuts, jute, lucerne, maize, rice, soybeans, tea, tobacco, and vegetables (aubergines, *Brassica*, *Capsicum*, cucurbit vegetables, *Phaseolus*, potatoes, sweet potatoes, and species of *Vigna*). Other hosts include ornamentals, wild plants, weeds, and shade trees [7]. *S. litura* have developed resistance to many commercially available pesticides, such as profenofos [8].

In order to have sustainable agriculture and improve public health, effective and appropriate pesticide management is necessary. Every year significant economic losses are reported, mainly because of damage to agricultural, forestry, and livestock production, caused by the persistence of insect pests; this fact makes adequate control of pests necessary. In this context, the scientific community continues a joint multidisciplinary effort to elucidate the mechanisms of resistance developed by pest organisms. One relevant contribution by Guo et al. (2017) is the development of a computational pipeline that uses AChE to detect resistance mutations of AChE in insect RNA-Sequencing data that facilitates the full use of large-scale genetic data obtained by next-generation sequencing [9]. A recent study by Brevik et al. (2018) reported that the median duration between the introduction of an insecticide and the first report of resistance was 66 generations (95% CI 60–78 generations) [10].

The prevalence of resistant insects is influenced by different factors that can be grouped into three categories: (1) Biological factors, such as generation time, number of offspring per generation, and migration; (2) genetic factors that include the frequency and dominance of the resistance gene, fitness of the resistance genotype, and the number of different resistance alleles; and (3) operational factors in which man intervenes, such as treatment, persistence, and insecticide chemistry, allusive to timing and dosage of insecticide application [11].

The term resistance to insecticides refers to a hereditary change in the sensitivity of a pest population that is reflected in recurrent failure to perform its insecticidal action, generating inadequate pest control [11,12,13]. There are several ways insects can become resistant: Behavioral resistance (resistant insects may detect or recognize a danger and avoid the toxin; they simply stop feeding), penetration resistance (resistant insects may absorb the toxin more slowly than susceptible insects), metabolic resistance (resistant insects may detoxify or destroy the toxin faster than susceptible insects), and altered target-site resistance (the toxin binding site becomes modified to reduce the insecticide’s effects); often, more than one of these mechanisms occurs at the same time [11].

According to the mechanisms of action of pesticides, more than 25 types of resistance have been identified and at least 55 types of chemical species [12,13]. One main group is acetylcholinesterase (AChE) inhibitors, which are divided into two subgroups, cataloged as carbamates and organophosphates; both affect the nervous system. AChE is a hydrolytic enzyme that acts on acetylcholine (ACh)—its natural substrate, a neurotransmitter—generating choline and acetic acid. In the presence of organophosphorus (OPs) pesticides, AChE is phosphorylated and, as a consequence, is inhibited [14,15]. 

**Figure 1:**
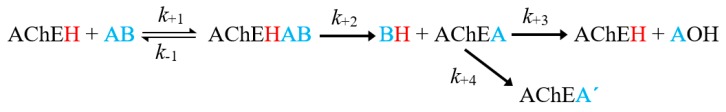
General scheme of the inhibition of acetylcholinesterase (AChE) by an organophosphorus (OP) inhibitor. Where AChEH is the free enzyme; AB, the OP molecule; AChEA’, the dealkylated form of the phosphorylated enzyme (AChEH) [14,15].

The inhibition process involves several stages, outlined in Figure 1. The first is the affinity of OPs for AChE, which determines the reversible inhibition of the enzyme (described by the affinity constant, *K*_a_ = k_+1_/k_−1_) [14]. The second is the phosphorylation constant (known as *k_+_*_2_ or *k*_p_), which governs the rate of formation of the stable covalent bond, causing permanent inhibition of the enzyme and the release of a leaving group (BH, Figure 1) [16]. The third is a hydrolysis reaction considered in homologous kinetic systems and widely studied in lines of research concerning self-reactivating cholinesterases [14,15]. The fourth process is known as the aging of phosphorylation [17,18]. It consists of the hydrolysis of one of the alkyl residues of the phosphate group bound to the active site, giving rise to a very stable complex, characterized by an acid group in the phosphoric center. The irreversibility of the reaction is established by the magnitude of the constant *k*_+4_ [18,19]. Regarding the ideal characteristics that a good OP pesticide must meet, at least four kinetic criteria are established in order to provide an optimal pesticide activity as well as safety for mammals: (1) *K*_a_ (pest) < *K*_a_ (mammals), (2) *K*_+2_ (pest) > *K*_+2_ (mammals), (3) *K*_+3_ (pest) < *K*_+3_ (mammals), and (4) *K*_+4_ (pest) > *K*_+4_ (mammals) [15]. 

The main interest of the research work is performed in an in silico study of the resistance to organophosphorus (OPs) pesticides associated with point mutations in AChE of Lepidoptera, using computational tools to elucidate the structural basis of the mechanism of resistance. The study considers the two forms of AChE present in Lepidoptera, known as form 1 acetylcholinesterase (AChE-1) and form 2 acetylcholinesterase (AChE-2), which present about 40% sequence identity. The OPs molecules evaluated include compounds identified in cases of resistance of Lepidoptera to OP insecticide, consulted in the Arthropod Pesticide Resistance Database (APRD) [20], as well as the database of the Insecticide Resistance Action Committee (IRAC) [21].

The study also includes two non-OPs molecules, acetylcholine (ACh)—a natural substrate of AChE—and a non-OP pesticide, psoralen. Psoralen is a naturally occurring furocoumarin, found in *Psoralea*, which is a genus in the legume family (Fabaceae). *Psoralea corylifolia* is an important medicinal plant that is used and widely studied in several traditional medicines to cure various diseases (e.g., anti-carcinogenic activity, anti-depressant activity, skin related problem/leukoderma, Alzheimer’s disease) [22,23]. 

## 2. Results

### 2.1. Computational Protocol

The implemented computational protocol consisted of three stages: (a) Modeling by homology using the SWISS-MODEL program [24,25], (b) point mutations performed with the FoldX program [26,27], and (c) molecular docking using Autodock 4.2 [28]. The AChE molecular models, as well as the respective modified models, were constructed satisfactorily (see details about results for Homology Modeling in Table A1, Appendix A). The quality of the models built by homology modeling was analyzed using the multiple 3D alignment option provided by the PDBeFold service (summary showed in Table 1) [29]. In this 3D alignment analysis, the structures that were used as templates for constructed models were used as a reference, PDB 5YDJ (AChE of *Anopheles gambiae*) and 1DX4 (AChE of *Drosophila melanogaster*) for AChE-1 and AChE-2 enzymes, respectively.

### 2.2. Molecular Docking of OPs on AChE Enzymes

**Figure 2:**
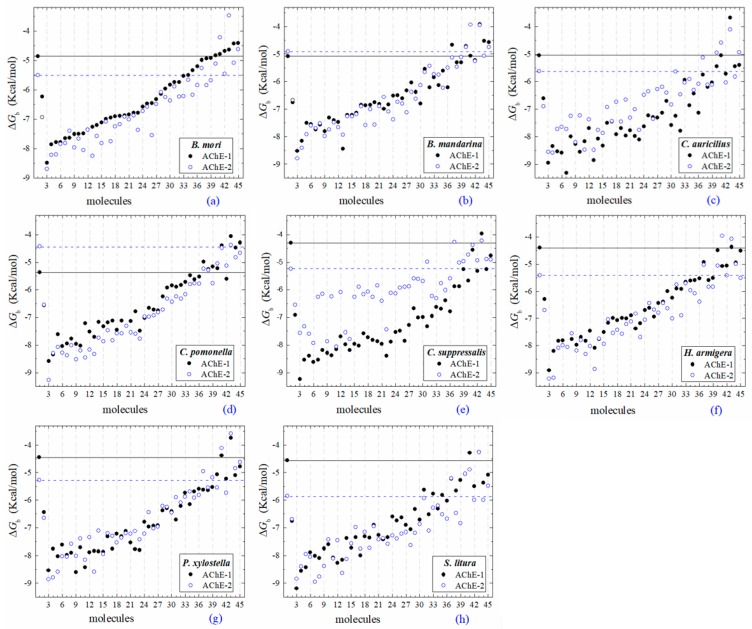
Molecular docking results of AChEs (AChE-1 and AChE-2, both wild type) in complex with molecules. AChE of the following organisms: *B. mori* (**a**), *B. mandarina* (**b**), *C. auricilius* (**c**), *C. pomonella* (**d**), *C. suppressalis* (**e**), *H. armígera* (**f**), *P. xylostella* (**g**), and *S.* *litura* (**h**). The molecules evaluated are the following: 1, acetylcholine; 2, psoralen; the OPs molecules: 3, leptophos (32c); 4, cyanofenphos (28c); 5, tetrachlorvinphos (39c); 6, phosalone (14c); 7, chlorfenvinphos (42c); 8, profenofos (37c); 9, dialifos (29c); 10, bromophos-ethyl (27c); 11, isoxathion (31c); 12, prothiofos (36c); 13, EPN (21c); 14, azinphos-methyl (3c); 15, phoxim (43c); 16, sulprofos (38c); 17, diazinon (5c); 18, chlorpyrifos (4c); 19, pirimiphos-methyl (34c); 20, bromophos (23c); 21, quinalphos (30c); 22, phosmet (17c); 23, triazophos (35c); 24, ronnel (16c); 25, carbophenothion-methyl (20c); 26, phenthoate (22c); 27, chlorpyrifos-methyl (26c); 28, mephosfolan (18c); 29, cyanophos (25c); 30, parathion (1c); 31, ethion (9c); 32, fenitrothion (33c); 33, dioxabenzofos (24c); 34, methidathion (19c); 35, malathion (10c); 36, parathion-methyl (12c); 37, disulfoton (8c); 38, dicrotophos (41c); 39, naled (13c); 40, dimethoate (7c); 41, trichlorfon (15c); 42, mevinphos (40c); 43, methamidophos (11c); 44, dichlorvos (6c); and 45, acephate (2c). Δ*G*_b_, the estimated energy of binding. Continuous line shows the energy threshold recorded in AChE-1^wild type^-ACh complex. Dotted line shows the energy threshold in AChE-2^wild type^-ACh complex. Docking was performed using the AutoDock4.2 program.

**Figure 3:**
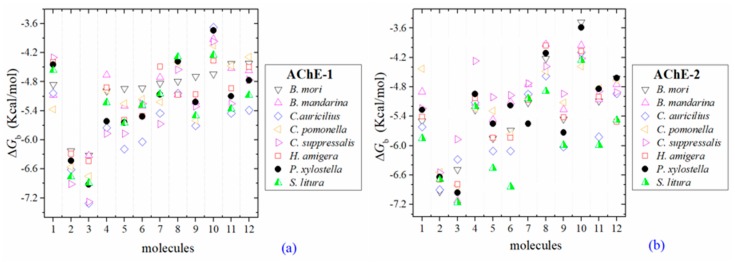
Predicted Δ*G*_b_ results of the low inhibition score of OPs molecules in AChE-1 (**a**) and AChE-2 (**b**), both wild type. Δ*G*_b_, estimated free energy of binding; molecules: 1, acetylcholine; 2, psoralen; 3, chlorpyrifos-methyl; 4, disulfoton; 5, dicrotophos; 6, naled; 7, dimethoate; 8, trichlorfon; 9, mevinphos; 10, methamidophos; 11, dichlorvos; and 12, acephate.

**Figure 4:**
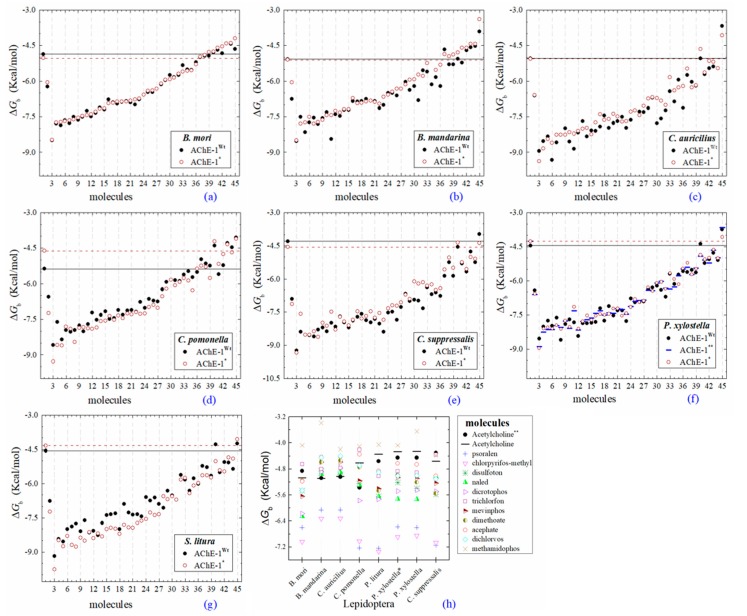
Predicted Δ*G*_b_ results of OPs molecules in AChE-1 (^Wt^, wild type, full circles in **a**–**g**, and, *, modified, empty circles in **a**–**g**). Δ*G*_b_, estimated free energy of binding; point mutations on AChE-1: A201S, G227A, and L452S to *B. mori* (**a**) and to *B. mandarina* (**b**); A201S to *C. auricilius* (**c**) and to *C. suppressalis* (**e**); F290V to *C. pomonella* (**d**); A201S, G227A, and F290A to *P. xylostella* (**f**) and *S. litura* (**g**); A201S and G227A to *P. xylostella* * (**f**); **, evaluated in AChE-1 wild type. The molecules evaluated are the following: 1, acetylcholine; 2, psoralen; the OPs molecules: 3, 32c; 4, 39c; 5, 28c; 6, 42c; 7, 14c; 8, 29c; 9, 37c; 10, 27c; 11, 21c; 12, 31c; 13, 36c; 14, 43c; 15, 3c; 16, 35c; 17, 4c; 18, 5c; 19, 23c; 20, 30c; 21, 34c; 22, 38c; 23, 17c; 24, 16c; 25, 20c; 26, 22c; 27, 26c; 28, 18c; 29, 25c; 30, 33c; 31, 1c; 32, 9c; 33, 10c; 34, 19c; 35, 24c; 36, 12c, parathion-methyl; 37, 8c, disulfoton; 38, 13c, naled; 39, 41c, dicrotophos; 40, 15c, trichlorfon; 41, 40c, mevinphos; 42, 7c, dimethoate; 43, 2c, acephate; 44, 6c, dichlorvos; and 45, 11c, methamidophos. In **a**–**g**, a continuous line shows the energy threshold recorded in the AChE-1^wild type^-ACh complex and a dotted line shows the energy threshold in the AChE-1^modified^-ACh complex. (**h**) Results of the ten OPs with lower inhibition score recorded (Δ*G*_b_^acetylcholine^ < Δ*G*_b_^OPs^) in **a**–**g**: These OPs molecules are 2c, 6c, 7c, 8c, 11c, 12c, 13c, 15c, 40c, and 41c. Δ*G*_b_, the estimated energy of binding. Docking was performed using the AutoDock4.2 program.

All the predicted Δ*G*_b_ from the docking of 43 OPs molecules (in addition to acetylcholine and psoralen molecules) on AChE enzymes of Lepidoptera (*B. mandarina*, *B. mori*, *C. auricilius*, *C. suppressalis*, *C. pomonella*, *H. armígera, P. xylostella*, and *S. litura*) are presented in three sections: (1) AChE-1 and AChE-2, both wild type, (2) modified AChE-1, and (3) modified AChE-2. In order to carry out an analysis of results, all the predicted Δ*G*_b_ are presented graphically (Figure 2, Figure 3 and Figure 4, respectively); the molecules evaluated are plotted against the estimated free binding energy (predicted Δ*G*_b_) obtained. In each section, the order of the molecules is presented according to the predicted Δ*G*_b_ recorded in *B. mori* (e.g., Figure 2a, Figure 3a and Figure 4a).

### 2.3. Docking of OPs Molecules on AChE-1 and AChE-2 (Wild Type)

Three global assessments of AChE wild type docking results are as follows: (1) The natural substrate molecule, acetylcholine (ACh) records values of −5.3 < Δ*G*_b_ < −4.30 Kcal/mol in complex with AChE-1, and in complex with AChE-2 −5.85 < Δ*G*_b_ < −4.43 Kcal/mol. (2) Psoralen (non-OP molecule) records a narrow range of Δ*G*_b_ values −6.94 < Δ*G*_b_ < −6.23 Kcal/mol, in complex with both AChE enzymes. (3) In the evaluation of the AChE enzymes (AChE-1 and AChE-2 wild type) by docking of OP (shown in Figure 2a–h), 33 OPs were identified that registered Δ*G*_b_^acetylcholine^ > Δ*G*_b_^OPs^, lower Δ*G*_b_ values with respect to the Δ*G*_b_ value recorded for ACh. These molecules were leptophos (32c), cyanofenphos (28c), tetrachlorvinphos (39c), phosalone (14c), chlorfenvinphos (42c), profenofos (37c), dialifos (29c), bromophos-ethyl (27c), isoxathion (31c), prothiofos (36c), O-ethyl O-(4-nitrophenyl) phenylphosphonothioate known as EPN (21c), azinphos-methyl (3c), phoxim 43c), sulprofos (38c), diazinon (5c), chlorpyrifos (4c), pirimiphos-methyl (34c), bromophos (23c), quinalphos (30c), phosmet (17c), triazophos (35c), ronnel (16c), carbophenothion-methyl (20c), phenthoate (22c), chlorpyrifos-methyl (26c), mephosfolan (18c), cyanophos (25c), parathion (1c), ethion (9c), fenitrothion (33c), dioxabenzofos (24c), methidathion (19c), and malathion (10c). Then 10 OPs were identified that register Δ*G*_b_^acetylcholine^ ≤ Δ*G*_b_^Ops^, that is, to register a binding energy with a magnitude equal to or greater than the natural substrate: These OPs were parathion-methyl (12c), disulfoton (8c), dicrotophos (41c), naled (13c), dimethoate (7c), trichlorfon (15c), mevinphos (40c), methamidophos (11c), dichlorvos (6c), and acephate (2c). In Figure 3, only the predicted Δ*G*_b_ results of ACh, psoralen, and the 10 OPs (molecules: 2c, 6c, 7c, 8c, 11c, 12c, 13c, 15c, 40c, and 41c) with the highest recorded predicted Δ*G*_b_ are shown.

### 2.4. Docking of OPs Molecules on Modified AChE-1

**Figure 5:**
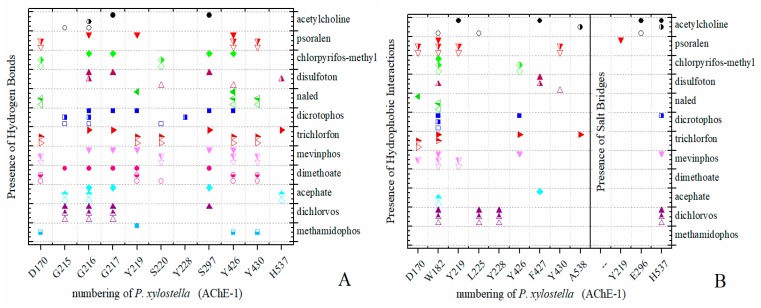
PLIP (Protein-Ligand Interaction Profiler) results in AChE-1 of *P. xylostella*. Identification of noncovalent interactions; (**A**) hydrogen bonds, (**B**) hydrophobic interactions and salt bridges. Full symbol, AChE-1 wild type; empty symbol, modified AChE-1 by A201S and G227A; half full symbol, modified AChE-1 by A201S, G227A, and F290A.

Predicted Δ*G*_b_ results of the AChE-1 wild type and modified AChE-1 enzymes are shown in Figure 4a–g. A global assessment is that 33 OPs in complex with modified AChE-1 (molecules: 1c, 3c, 4c, 5c, 9c, 10c, 14c, 16c, 17c, 18c, 19c, 20c, 21c, 22c, 23c, 24c, 25c, 26c, 27c, 28c, 29c, 30c, 31c, 32c, 33c, 34c, 35c, 36c, 37c, 38c, 39c, 42c, and 43c) register Δ*G*_b_ values below the threshold registered for AChE-1 in complex with acetylcholine (i.e., Δ*G*_b_ of AChE-1 wild type-ACh complex > Δ*G*_b_ of modified AChE-1-OP complex) this is Δ*G*_b_^acetylcholine^ > Δ*G*_b_^OPs^. In Figure 4h, only the results of ACh, psoralen, and the 10 OPs (molecules: 2c, 6c, 7c, 8c, 11c, 12c, 13c, 15c, 40c, and 41c) with the highest recorded predicted Δ*G*_b_ are shown, which is a lower inhibition score recorded (Δ*G*_b_^acetylcholine^ ≤ Δ*G*_b_^Ops^). Docking results of the 11c molecule, methamidophos, recorded a Δ*G*_b_^ACh^ < Δ*G*_b_^11c^ in the AChE-1 (both wild type and modified) on the seven evaluated organisms. In the AChE-1 (both wild type and modified) of *P. liture* and *P. xylostella,* nine OPs molecules (molecules: 2c, 6c, 7c, 8c, 12c, 13c, 15c, 40c, and 41c) presented a similar trend Δ*G*_b_^Ach^ > Δ*G*_b_^OPs^. But in AChE-1 of *B. mandarina* and *C. auricilius*, these nine OPs molecules presented Δ*G*_b_^ACh^ < Δ*G*_b_^OPs^. To identify the non-covalent interactions between the AChE-1 enzymes of *P. xylostella* and their ligands, the respective molecular models (presented in Figure 4) were analyzed with PLIP (Protein-Ligand Interaction Profiler) [30]; data shown in Figure 5.

### 2.5. Docking of OPs Molecules on Modified AChE-2

**Figure 6:**
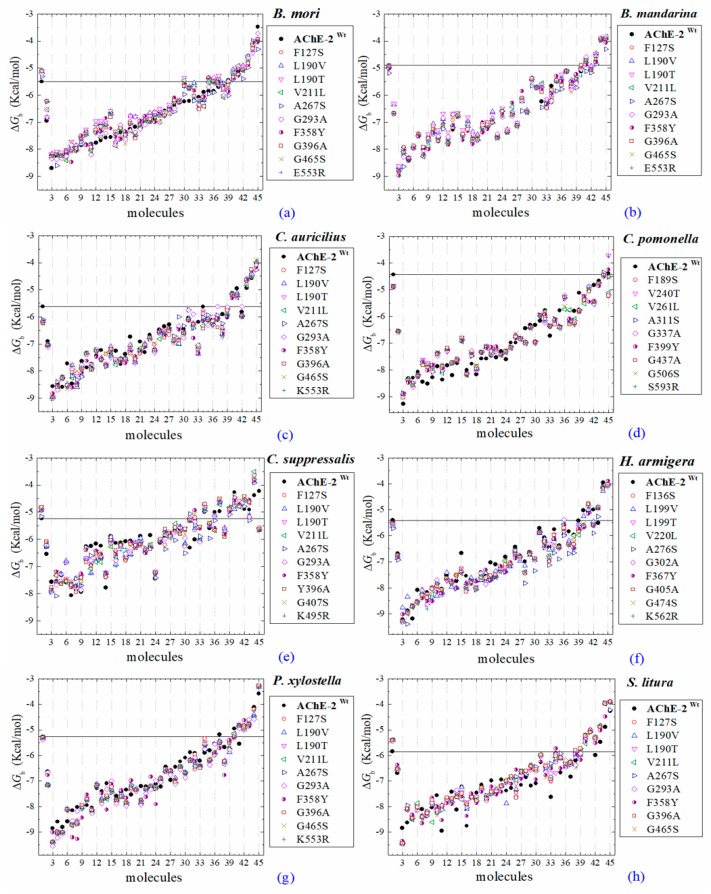
Molecular docking results of AChEs (AChE-2 wild type and AChE-2 modified) in complex with molecules. AChE-2 (^wt^, wild type enzyme) of the following organisms: *B. mori* (**a**), *B. mandarina* (**b**), *C. auricilius* (**c**), *C. pomonella* (**d**), *C. suppressalis* (**e**), *H. armígera* (**f**), *P. xylostella* (**g**), and *S.* *litura* (**h**). The point mutations on AChE-2 are indicated in box, respectively. The molecules evaluated are the following: 1, acetylcholine; 2, psoralen; the OPs: 3, 32c; 4, 21c; 5, 28c; 6, 39c; 7, 31c; 8, 29c; 9, 14c; 10, 43c; 11, 42c; 12, 5c; 13, 27c; 14, 3c; 15, 22c; 16, 37c; 17, 35c; 18, 36c; 19, 4c; 20, 34c; 21, 38c; 22, 30c; 23, 23c; 24, 17c; 25, 16c; 26, 20c; 27, 26c; 28, 1c; 29, 25c; 30, 33c; 31, 24c; 32, 10c, malathion; 33, 18c, mephosfolan; 34, 9c, ethion; 35, 12c, parathion-methyl; 36, 41c, dicrotophos; 37, 13c, naled; 38, 19c, methidathion; 39, 40c, mevinphos; 40, 8c, disulfoton; 41, 7c, dimethoate; 42, 6c, dichlorvos; 43, 2c, acephate; 44, 15c, trichlorfon; and 45, 11c, methamidophos. Δ*G*_b_, the estimated energy of binding. A continuous line shows the energy threshold recorded in AChE-2^wild type^-ACh complex. Docking was performed using the AutoDock4.2 program.

Docking results of the molecules evaluated on AChE-2 (wild type and modified) showed some appreciable variations in the magnitude of Δ*G*_b_ registered among the enzymes of the 8 evaluated organisms (see Figure 6). The global assessment is (1) that 29 OPs were identified that register Δ*G*_b_*^ach^* < Δ*G*_b_^OPs^ (molecules: 1c, 3c, 4c, 5c, 14c, 16c, 17c, 20c, 21c, 22c, 23c, 24c, 25c, 26c, 27c, 28c, 29c, 30c, 31c, 32c, 33c, 34c, 35c, 36c, 37c, 38c, 39c, 42c, and 43c) (see Figure 6a–h), (2) that six OPs were identified that register Δ*G*_b_^ACh^ > Δ*G*_b_^OPs^ (2c, acephate, 6c, dichlorvos, 7c, dimethoate, 8c, disulfoton, 11c, methamidophos, and 15c, trichlorfon, except for AChE-2 that were only 2c, 11c, and 15c), and (3) the another 8 compounds (9c, ethion, 10c, malathion, 12c, parathion-methyl, 13c, naled, 18c, mephosfolan,, 19c, methidathion, 40c, mevinphos, and 41c, dicrotophos) registered a different magnitude in the AChE enzymes evaluated. Predicted Δ*G*_b_ results of the AChE-2 wild type and modified AChE-2 enzymes are shown in Figure 6a–h.

## 3. Discussion

### 3.1. Construction of Molecular Models

The molecular models of the respective AChE enzymes are reliable. In the evaluation of their quality, they register acceptable scores both in their construction (Table A1, Appendix A) and in the 3D alignment, with respect to the reference crystallographic structure (Table 1). The certainty of the structural predictions is based on the quality of the molecular models (e.g., root-mean-square deviation (RMSD) and quality of alignment (Q^score^)). In our study, the constructed models are reliable and reproduce the experimental reference evidence.

### 3.2. Docking Results of Acetylcholine Evaluations on AChE

The docking results of the AChE–ACh complex, obtained in the evaluation of AChE-1, form 1 of AChE, and AChE-2, form 2 of AChE (both wild type and modified enzymes), are highlighted below.

(A) AChE-1 and AChE-2 (both wild type enzymes), the energy threshold registered for ACh shows a difference in magnitude between Δ*G*_b_^AChE-1 wild type^ and Δ*G*_b_^AChE-2 wild type^ (see Figure 6), the exception is AChE of *B. mandarina* (Figure 2b) (the energies recorded to AChE-2-ACh complexes were equal in magnitude, Δ*G*_b_^AChE-1 wild type^ = Δ*G*_b_^AChE-2 wild type^). The result obtained in AChE of *B. mandarina* suggests that there is no difference in the affinity of AChEs (Δ*G*_b_^AChE-2 wild type^ and Δ*G*_b_^AChE-2 wild type^) for the substrate. Whereas, in the other systems, a difference in affinity between AChE and ACh is predicted, which could be confirmed by catalytic activity studies of the enzymes.

(B) AChE-1^wild type^ and AChE-1^modified^, as seen in Figure 4, six studied systems (*B. mori—*Figure 4a, *B. mandarina*—Figure 4b, *C. auricilius*—Figure 4c, *C. suppressalis—*Figure 4e, *P. xylostella*—Figure 4f, and *S. litura*—Figure 4g) showed that no difference or significant deviations was observed between the energy threshold registered for ACh in Δ*G*_b_^AChE-1 wild type^ and Δ*G*_b_^AChE-1 modified^. Only AChE-1 of *C. pomonella* (Figure 4d) in complex with ACh recorded an absolute relative energy value near to 0.7 Kcal/mol. This is a Δ*G*_b_^AChE-1 modified^ greater than the Δ*G*_b_^AChE-1 wild type^. This result suggests a decrease in affinity for the substrate *ace*. For the other AChE-1–ACh complexes analyzed, there was no variation between the two enzymes (AChE-1^wild type^ and AChE-1^modified^). This result suggests that the mutations evaluated in AChE-1 do not affect the affinity of *ace*.

(C) AChE-2^wild type^ and AChE-2^modified^, all evaluations of ACh in AChE-2 did not record a variation between Δ*G*_b_^AChE wild type^ and Δ*G*_b_^AChE modified^; the energy thresholds of ACh in AChE-2 evaluations do not register a variation (Figure 6).

### 3.3. Docking Results of Psoralen Evaluations on AChE

The docking results of the AChE–psoralen complex recorded a score −6.8 Kcal/mol < Δ*G*_b_ < −6.0 Kcal/mol. This result was consistent in all the docking evaluations on AChE (AChE-1 and AChE-2, both wild type and modified with slight variations (see Figure 2a–h, Figure 4a–g and Figure 6a–h)). The results obtained suggest that the specific modifications made to AChE do not affect the recorded energy. Results presented in Figure 5 supports this assertion; the profile of interactions recorded in the AChE-1 of *P. xylostella* in complex with psoralen shows the presence of hydrogen bonds (recorded in residue Y426) and hydrophobic interactions (recorded in residue W182), present in both AChE-1^wild type^ and AChE-1^modified^. The results of the energy predictions of the enzyme AChE of Lepidoptera in the study of psoralen are promising for an in vitro study, mainly for two reasons: The first is its characteristic of being a natural molecule (no-OP) with inhibitory activity to AChE, and the second, is the scores psoralen obtained in the binding to AChE register a Δ*G*_b_ < −6.0 Kcal/mol in complex with both AChE enzymes (AChE-1 and AChE-2) of the Lepidoptera organisms studies here. 

### 3.4. Resistance-Associate Mutation on AChE

**Figure 7:**
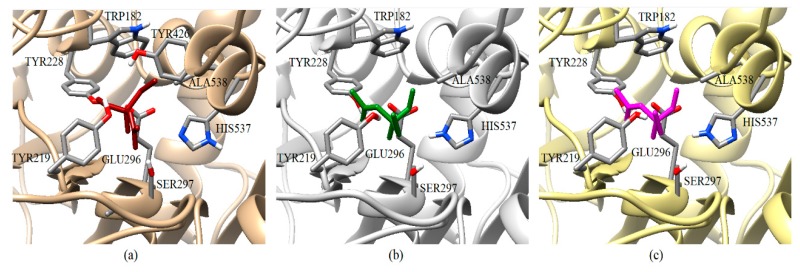
Graphical representation of the acephate binding on AChE-1 of *P. xylostella*. Spatial localization of acephate structure on the AChE-1 enzyme as a result of docking is shown in three molecular models: Conformation acquired on AChE-1 wild type, acephate is chain in red (**a**), conformation acquired on modified AChE-1 (A201S and G227A, and -A201S, G227A, and F290A, respectively), the acephate is chain in green and magenta (**b** and **c**, respectively). The main interactions are shown in Figure 5. The location of SER297 is shown, which is an important residue for the effect of enzyme phosphorylation. HIS537 in AChE-1 of *P. xilostella* is stabilized by GLU296 and GLU423. The p*K*a values of ionizable residues could be predicted with PROPKA [31].

A review of the cases of registered incidences of resistance of Lepidoptera exclusively to the mechanism of action (MoA) of AChE inhibitors by OP (source: www.pesticideresistance.org, consulted in 2018-August) resulted in the top nine resistant Lepidoptera species shown in Table 2. There is a great consistency between the results presented in Figure 4 and Figure 5 and the information contained in Table 2. Eight OPs (chlorpyrifos-methyl (26c), disulfoton (8c), dicrotophos (41c), trichlorfon (15c), mevinphos (40c), dimethoate (7c), acephate (2c), and dichlorvos (6c), in complex with AChE-1 wild type of *P. xylostella*, presented a hydrogen bond between S297 and O3 from ligand (OP insecticide). This interaction was very important in the effect of irreversible inhibition (Figure 1). In the respective modified enzymes, this interaction is absent—AChE-1 was modified by A201S and G227A and by A201S, G227A, and F290A. Predicted Δ*G*_b_ results of AChE enzymes in complex with OPs molecules recorded an consistent energy values in the majority of the evaluations, reflected in the profile of the graphics presented in Figure 2a–h, Figure 4a–g and Figure 6a–h, according to the chemical composition of the OP molecule. However, the energy predictions between wild and modified enzymes (AChE-1 and AchE-2) do not show a relevant effect on the recorded magnitude; that is, the proposed mutations do not give rise to an enzyme insensitivity. Molecular models of modified AChE (with point mutations) provide information on predicted Δ*G*_b_, in addition to the possible conformation of the interaction mode. Docking results also showed a different location of the binding site of OPs on the enzyme, as a consequence of a change in the electronic atmosphere caused by the point mutation (e.g., acephate (2c) in complex with AChE-1 of *P. xylostella*, Figure 7). 

The contributions of the present study about modified AChE-2 of Lepidoptera in complex with OP are mainly two: (1) Docking results give a configuration of the most probable spatial orientation of two interacting molecules (AChE enzyme and OP pesticide), and (2) a predicted Δ*G*_b_. Regarding the predicted Δ*G*_b_ results obtained from the evaluations in wild type and modified AChE-2, the results are not conclusive, regarding the effect of the proposed mutations in the direction of generating insensitivity of the enzyme. Only small variations were obtained in the magnitude of the predicted energy, which could be due to the variation of the estimate.

## 4. Materials and Methods

### 4.1. Homology Modeling

The AChE sequences (form 1 known as AChE-1 and form 2 known as AChE-2) of the nine lepidopterous organisms were retrieved from GenBank of the National Centre for Biotechnology Information [32]. The accession numbers of AChE-1 and AChE-2 were the following: EU262633.2 and EU262632.2 of *B. mandarina*; NM_001043915.1 and NM_001114641.1 of *B. mori*; KF574430.1 and KF574431.1 of *C. auricilius*; EF453724.1 and EF470245.1 of *C. suppressalis*; DQ267977.1 and DQ267976.1 of *C. pomonella*; JF894118.1 and JF894119.1 of *H. armigera*; JQ085429.1 and AY061975.1 of *P. xylostella*; AQQ79918.1 and AQQ79919.1 of *S. litura*; and AGK44160.1 (AChE-1) of *S. frugiperda*. Therefore, the molecular models of AChEs were constructed by homology using the SWISS-MODEL program, operating in the search-templates mode, followed by a user-template mode [22,25]. The PDB structures of AChE that recorded the best score (Appendix A) were selected to be used as templates.

### 4.2. Point Mutations on the AChE Structures

**Figure 8:**
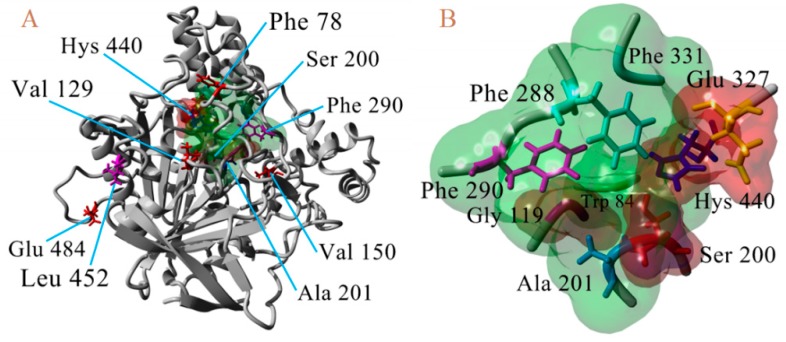
3D atomic model of AChE, numbering of *T. californica*. (**A**). Location of waste susceptible to proposed point mutations. (**B**) Proximity of catalytic triad (Ser^200^, Hys^440^, and Glu^327^).

Once the 3D models of the wild type AChE enzyme were constructed, we proceeded to perform point mutations, previously described as possibly responsible for diverse resistance to carbamates and organophosphorus compounds in many insect species [33]. The AChE-2 structures of *B. mandarina*, *B. mori*, *C. auricilius*, *C. suppressalis*, *C. pomonella*, *H. armígera, P. xylostella*, and *S.* *litura* were modified in eight residues considered the most important mutations in Diptera organisms [9,34,35] (F78S, L(V)129V/T, V150L, A201S, G227A, F290Y, G328A, and G396S, A(K, S)484R corresponding AChE numbering of *T. californica*) with the FoldX program, using the FoldX Tool BuildModel [26,27]. In addition, molecular models of AChE-1 were generated to construct the modified enzyme by resistance-associated mutations in lepidopterous organisms [9]: A201S, G227A and L452S to *B. mandarina* and to *B. mori*; A201S to *C. auricilius*; A201S to *C. suppressalis*; F290V to *C. pomonella*; A201S, G227A and F290A to *P. xylostella* and *S. litura*, corresponding to the AChE numbering of *T. californica*; in Figure 8, a 3D representation is presented.

### 4.3. Ligands Used in Docking

All OPs molecules were obtained from the PubChem database [36,37]. The identification codes (CID) of the compounds were the following: CID991 (parathion, 1c), CID1982 (acephate, 2c), CID2268 (azinphos-methyl, 3c), CID2730 (chlorpyrifos, 4c), CID3017 (diazinon, 5c), CID3039 (dichlorvos, 6c), CID3082 (dimethoate, 7c), CID3118 (disulfoton, 8c), CID3286 (ethion, 9c), CID4004 (malathion, 10c), CID4096 (methamidophos, 11c), CID4130 (parathion-methyl, 12c), CID4420 (naled, 13c), CID4793 (phosalone, 14c), CID5853 (trichlorfon, 15c), CID9298 (ronnel, 16c), CID12901 (phosmet, 17c), CID13707 (mephosfolan, 18c), CID13709 (methidathion, 19c), CID13721 (carbophenothion-methyl, 20c), CID16421 (EPN, 21c), CID17435 (phenthoate, 22c), CID16422 (bromophos, 23c), CID19657 (dioxabenzofos, 24c), CID17522 (cyanophos, 25c), CID21803 (chlorpyrifos-methyl, 26c), CID20965 (bromophos-ethyl, 27c), CID25669 (cyanofenphos, 28c), CID25146 (dialifos, 29c), CID26124 (quinalphos, 30c), CID29307 (isoxathion, 31c), CID30709 (leptophos, 32c), CID31200 (fenitrothion, 33c), CID34526 (pirimiphos-methyl, 34c), CID32184 (triazophos, 35c), CID36870 (prothiofos, 36c), CID38779 (profenofos, 37c), CID37125 (sulprofos, 38c), CID5284462 (tetrachlorvinphos, 39c), CID5355863 (mevinphos, 40c), CID5371560 (dicrotophos, 41c), CID5377784 (chlorfenvinphos, 42c), and CID9570290 (phoxim, 43c). In addition, the natural substrate molecule CID187 (acetylcholine (ACh)) and a non-OPs pesticide (CID6199, psoralen) were included as a control in docking evaluations.

### 4.4. Verification of Computational Methodology

The computational methodology was verified by comparing our results of the binding energy (Δ*G*_b_) predicted with the docking results reported previously (see Appendix B) by Somani et al. (2015) to AChE of humans in complex with psoralen (PDB 1EVE was used) [22] and Ranjan et al. (2018) to AChE of humans in complex with several ligands (PDB 3LII was used) [38]; phosalone (CID6199), dimefox (CID8264), dichlorvos (CID5371560), phoxim ethyl phosphonate (CID6507160), heptenophos (CID62773), and methamidophos (CID4096). These molecules were obtained from the PubChem database [36,37]. The docking evaluation was carried out as described in the molecular docking section.

### 4.5. Molecular Docking of Ligands Targeting AChE Enzymes

In order to perform the molecular docking, all the molecular models of the AChE (wild type and modified by point mutations) were prepared in the UCSF Chimera program using the tool dockPrep with standard protocol [39] and saved in PDBQT format, and all ligands were saved in the mol2 format, according to the standard protocol. For each protein structure (AChE-1 and AChE-2) a GridBox was built with a dimension of X = 18.8 Å, Y = 18.8 Å and Z = 18.8 Å, using the MGTool program [28]. The coordinates of the center of the GridBox used were X_1_ = −59.62, Y_1_ = 58.834, and Z_1_ = 16.476. The docking was performed using the AutoDock4.2 program [28], which was installed on a Linux Mint 17.3 operating system implemented with a 3.4 GHz Intel Core i7 processor and 23.5 GB RAM. All molecular models were analyzed with PLIP (Protein-Ligand Interaction Profiler) [30].

## 5. Conclusions

The Lepidoptera family has an important economic impact in the world. However, resistance to insecticides is a very common problem in different countries. This resistance is mainly associated with punctual mutations; therefore, a model that helps us know how this change affects the activity of the insecticides is necessary to obtain or apply better control strategies. Based on the results in this study, the proposed mutations are not associated with the presence of insensitivity to the enzyme. However, the mutations evaluated in the AChE-1 and AChE-2 structures of enzymes do not affect in any way (there is no regularity of change or significant deviations) the values of the binding energy (Δ*G*_b_) recorded in the AChE-OPs complexes. Therefore, it is assumed that the proposed mutations confer resistance due to an inadequate steric interaction that prevents a phosphorylation reaction of the enzyme by the OP molecule and, therefore, is an irreversible inhibition.

## Figures and Tables

**Table 1:** Alignment results (performed with PDBeFOLD web server [29]). N^res^, number of aligned residues; %^SI^, % Sequence Identity; root-mean-square deviation (RMSD); quality of alignment (Q^score)^, with 1 being the highest score.

Model	AChE-1				AChE-2			
	N^res^	%^SI^	RMSD	Q^score^	N^res^	%^SI^	RMSD	Q^score^
*B. mandarina*	539	71.8	0.4837	0.935	551	62.9	0.3079	0.808
*B. mori*	539	72.0	0.4837	0.935	551	62.9	0.3079	0.808
*C. auricilius*	540	71.0	0.3956	0.941	548	63.1	0.3553	0.810
*C. suppressalis*	540	71.0	0.4593	0.936	493	62.7	0.5486	0.883
*C. pomonella*	551	44.1	1.3909	0.772	540	40.4	1.4337	0.678
*H. armígera*	540	72.0	0.3577	0.944	551	63.1	0.3514	0.806
*P. xylostella*	541	71.6	0.3695	0.942	551	63.3	0.3626	0.805
*S. litura*	540	72.0	0.3354	0.946	551	63.3	0.3784	0.804

**Table 2:** Top nine Lepidoptera organisms involved in resistance to OPs. ^£^ Studied in this work * Period of 2010–2016 [20].

Genus Species	# Cases:		OPs Insecticide
*Plutella xylostella* ^£^	862	279 *	1c, 2c*, 4c*, 5c, 6c, 7c, 10c, 11c, 12c, 13c, 15c, 18c, 19c, 21c, 22c, 24c, 26c, 28c, 28c, 29c, 30c, 31c, 32c, 33c, 34c, 35c, 36c, 37c, 40c*, and 43c
*Helicoverpa armígera* ^£^	856	129 *	3c, 4c*, 10c, 12c, 30c, 31c, 37c*, and 43c
*Spodoptera litura* ^£^	644	251 *	4c*, 5c, 6c, 10c, 15c, 30c, 35c, 37c*, 42c, and 43c*
*Spodoptera exigua*	525	303 *	4c*, 12c, 30c, and 37c*
*Cydia pomonella* ^£^	193	46 *	1c, 3c*, 4c*, 12c*, and 17c
*Chilo suppressalis* ^£^	148	79 *	1c, 4c, 5c, 11c, 15c, 21c, 33c, and 35c
*Earias vittella*	128	32 *	4c, 35c, and 37c
*Spodoptera frugiperda* ^£^	125	57 *	2c*, 4c, 5c, 6c, 10c, 12c,15c, and 38c
*Heliothis virescens*	120	---	1c, 12c, 16c, 17c, 27c, and 38c

**Figure A1:**
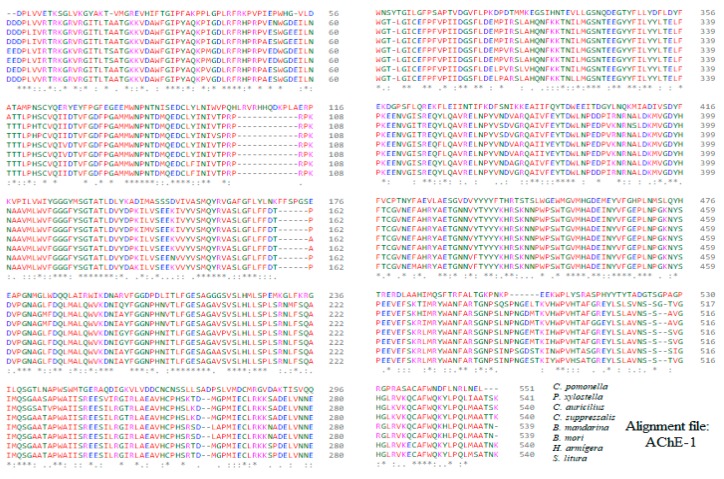
Alignment of the sequences of the constructed molecular models of AChE-1 (form 1 acetylcholinesterase).

**Figure A2:**
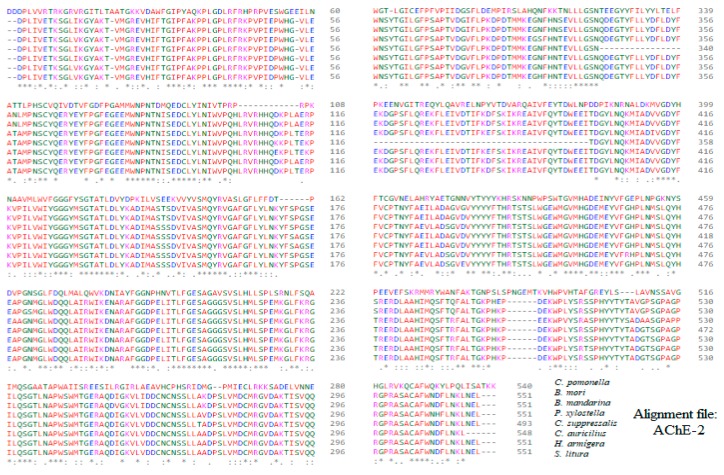
Alignment of the sequences of the constructed molecular models of AChE-2 (form 2 acetylcholinesterase).

## Abbreviations

AChE	acetylcholinesterase
AChE-1^wild type^	form 1 acetylcholinesterase, wild type, without mutations
AChE-1^modified^	form 1 acetylcholinesterase, modified, with puntual mutations
AChE-2 ^wild type^	form 2 acetylcholinesterase, wild type, without mutations
AChE-2 ^modified^	form 2 acetylcholinesterase, modified, with puntual mutations
OP	organophosphorus
Δ*G*_b_	binding energy
RMSD	root-mean-square deviation
Q^score^	quality of alignment

## Appendix A

### Results for the Homology Modeling and Multiple Alignment of AChE Enzymes

Once the sequences of AChE enzymes of Lepidoptera indicated in Section 4 were retrieved from The National Center for Biotechnology Information [32], we proceeded to construct the molecular models by homology using the SWISS-MODEL program (operating in search-templates mode, followed by user-template mode) [24,25]. The best recorded score was 5YDJ (AChE of *Anopheles gambiae*) and 1DX4 (AChE of *Drosophila melanogaster*) for AChE-1 and AChE-2, respectively. Data sheets of the SWISS-MODEL homology modelling report is presented in Table A1.

**Table A1:** Model building report of AChE (AChE-1 and AChE-2, both wild type) of Lepidoptera organisms [24,25].

AChE-1 Model	Template	Seq^I^	GMQE	QMEAN^Zs^	Seq^S^	Range/Covererage
*B. mandarina*	5YDJ	68.77	0.73	−1.64	0.52	103-642/0.83
*B. mori*	5YDJ	70.62	0.74	−1.60	0.53	103-642/0.80
*C. auricilius*	5YDJ	66.39	0.72	−1.70	0.51	114-653/0.88
*C. suppressalis*	5YDJ	67.46	0.71	−2.27	0.51	87-653/0.85
*C. pomonella*	5YDJ	66.99	0.72	−1.43	0.51	111-650/0.88
*H. armígera*	5YDJ	67.27	0.72	−1.45	0.51	115-654/0.87
*P. xylostella*	5YDJ	67.49	0.74	−1.47	0.51	98-638/0.90
*S. litura*	5YDJ	67.27	0.73	−1.42	0.51	114-653/0.88
**AChE-2 Model**	**Template**	**Seq^I^**	**GMQE**	**QMEAN**	**Seq^S^**	**Range/Covererage**
*B. mandarina*	1DX4	59.10	0.72	−2.38	0.49	53-603/0.87
*B. mori*	1DX4	60.18	0.74	−2.52	0.49	53-603/0.87
*C. auricilius*	1DX4	60.81	0.74	−2.51	0.50	53-600/0.86
*C. suppressalis*	1DX4	58.08	0.71	−3.00	0.48	53-542/0.84
*C. pomonella*	1DX4	60.72	0.74	−2.46	0.49	53-603/0.87
*H. armígera*	1DX4	60.36	0.73	−2.90	0.49	62-612/0.86
*P. xylostella*	1DX4	60.54	0.74	−2.67	0.49	53-603/0.87
*S. litura*	1DX4	60.54	0.74	−2.23	0.49	53-603/0.87

Sequence Identity (Seq^I^); Global Model Quality Estimation (GMQE); a comprehensive scoring function for model quality assessment (QMEAN^Zs^); Sequence Similarity (Seq^S^).

The parameters recorded in the evaluation of built models refer to the structural aspects linked to the template used. The Global Model Quality Estimation (GMQE) score is expressed in values of 0 to 1. Higher numbers indicate higher reliability, reflecting the expected accuracy of a model built with that alignment and template, as well as the coverage of the target. The comprehensive scoring function for model quality assessment (QMEAN^Zs^) score provides an estimate of the model on a global scale. Values recorded around zero indicate good agreement between the model structure and experimental structures of similar size, and score values of –4.0 or below indicate low quality models. The alignment of the sequences of the molecular models constructed are presented in Figure A1 (AChE-1) and Figure A2 (AChE-2). Alignments were executed with the Crustal Omega tool [40,41].

## Appendix B

### Verification of Computational Methodology

The verification of computational methodology consisted of reproducing results of Δ*G*_b_, previously reported by Ranjan et al. 2018 [38], Somani et al. 2015 [22], Chaudhry et al. 2013 [42], and Sharma et al. 2011 [43] approaches the interaction of OP with human AChE, where these authors use crystallographic structures to perform their analyses. In order to detect the variability in reproducibility in energy prediction, we evaluated 28 OPs molecules using our methodology (results not shown). We detected (performing 10 runs, in accordance with the docking protocol for the Autodoc 4.2) program variations in the determination of Δ*G*_b_ of 0.5 Kcal/mol; this parameter was obtained by analyzing the variation in the results of the evaluations of the 28 OPs.

The Δ*G*_b_ predictions obtained by docking with the purpose of comparing the computational protocol with other computational methodology reporters, in which six molecules on human AChE (PDB: 1EVE) were evaluated, provided results according to those previously reported [22,38] by other research groups (see Table A2). The docking study performed by Somani et al. (2015) and Ranjan et al. (2018) was carried out using the standard glide molecular docking protocol implemented within the Maestro molecular modeling suite by Schrodinger [22,38]. The results register a relative binding energy (ΔΔ*G*_b_) with a value up to −1.42 Kcal/mol. Considering our variation in the determination of Δ*G*_b_ (of 0.5 Kcal/mol), we suggest that the results are comparable.

**Table A2:** Data sheet of comparison of computational methodologies.

Ligands	Δ*G*_b_ *	Δ*G*_b_	ΔΔ*G*_b_
Psoralen	−6.84 ^a^	−6.97	0.13
Dimefox	−4.14 ^b^	−4.39	0.25
Dichlorvos	−4.47 ^b^	−5.52	1.05
Phoxim ethyl phosphonate	−6.25 ^b^	−7.33	1.08
Heptenophos	−5.66 ^b^	−6.83	1.17
Methamidophos	−5.87 ^b^	−4.45	−1.42

^a^, Somani et al., 2015 [22]; ^b^, Ranjan et al. (2018) [38]; Δ*G*_b_, predicted binding energy in Kcal/mol; *, previously reported; ΔΔ*G*_b_ = Δ*G*_b_* − Δ*G*_b_, relative binding energy.
